# Strategy to recognize and initiate treatment of chronic heart failure in primary care (STRETCH): a cluster randomized trial

**DOI:** 10.1186/1471-2261-14-1

**Published:** 2014-01-08

**Authors:** Evelien ES van Riet, Arno W Hoes, Alexander Limburg, Henk van der Hoeven, Marcel AJ Landman, Frans H Rutten

**Affiliations:** 1 Julius Center for Health Sciences and Primary care, University Medical Center Utrecht, PO Box 85500, 3508 AB, Utrecht, The Netherlands; 2 Diakonessenhuis Zeist, Zeist, The Netherlands

**Keywords:** Heart failure, Diagnosis, Treatment, Elderly, Primary care, Cluster randomized trial

## Abstract

**Background:**

Most patients with heart failure are diagnosed and managed in primary care, however, underdiagnosis and undertreatment are common. We assessed whether implementation of a diagnostic-therapeutic strategy improves functionality, health-related quality of life, and uptake of heart failure medication in primary care.

**Methods/Design:**

A selective screening study followed by a single-blind cluster randomized trial in primary care. The study population consists of patients aged 65 years or over who presented themselves to the general practitioner in the previous 12 months with shortness of breath on exertion. Patients already known with established heart failure, confirmed by echocardiography, are excluded. Diagnostic investigations include history taking, physical examination, electrocardiography, and serum N-terminal pro B-type natriuretic peptide levels. Only participants with an abnormal electrocardiogram or an N-terminal pro B-type natriuretic peptide level exceeding the exclusionary cutpoint for non-acute onset heart failure (> 15 pmol/L (≈ 125 pg/ml)) will undergo open-access echocardiography. The diagnosis of heart failure (with reduced or preserved ejection fraction) is established by an expert panel consisting of two cardiologists and a general practitioner, according to the criteria of the European Society of Cardiology guidelines.

Patients with newly established heart failure are allocated to either the 'care as usual’ group or the 'intervention’ group. Randomization is at the level of the general practitioner. In the intervention group general practitioners receive a single half-day training in heart failure management and the use of a structured up-titration scheme. All participants fill out quality of life questionnaires at baseline and after six months of follow-up. A six-minute walking test will be performed in patients with heart failure. Information on medication and hospitalization rates is extracted from the electronic medical files of the general practitioners.

**Discussion:**

This study will provide information on the prevalence of unrecognized heart failure in elderly with shortness of breath on exertion, and the randomized comparison will reveal whether management based on a half-day training of general practitioners in the practical application of an up-titration scheme results in improvements in functionality, health-related quality of life, and uptake of heart failure medication in heart failure patients compared to care as usual.

**Trial registration:**

ClinicalTrials.gov NCT01202006

## Background

Heart failure (HF) is an emerging epidemic in the elderly, causing high mortality rates, substantial loss in quality of life, and high healthcare costs [1]. The last two decades, progress in patient management, including multiple drug therapies and implantable devices, has improved prognosis of patients with heart failure with reduced ejection fraction (HF-REF) [2,3]. Poor compliance to and insufficient up-titration of evidence-based medication is a major contributor to hospital (re)admissions and loss of life years in these patients [4]. Angiotensin converting enzyme (ACE) inhibitors, angiotensin receptor blockers (ARBs) and beta-blockers did not show a significant beneficial effect in patients with heart failure with preserved ejection fraction (HF-PEF) [5], and their prognosis did not improve over the last years [6]. Currently, adequate treatment of hypertension and other comorbidities, control of heart rate in those with concurrent atrial fibrillation, and diuretics to control sodium and water retention are considered important in HF-PEF [4].

The majority of the elderly patients with HF is diagnosed and managed in primary care [7]. Unfortunately however, recognizing HF in the early stage is challenging, and echocardiography is not readily available in primary care. A diagnosis based on the signs and symptoms only, without echocardiography, results in both false-positive [8,9] and false-negative diagnoses [10]. Moreover, uncertainty about HF diagnosis and fear of side effects have led to underutilization of ACE-inhibitors and beta-blockers in patients with HF in primary care [11,12].

We suspect that especially in older persons presenting with shortness of breath to the general practitioner (GP), the prevalence of undetected HF is high. Therefore, we developed a selective screening strategy, including an open-access outpatient echocardiography facility. In addition we developed an easy to apply up-titration scheme for those newly diagnosed with HF-REF and HF-PEF. Our main objective is to assess whether such a targeted diagnostic-therapeutic approach improves current underdiagnosis and undertreatment.

### Key objectives

– To determine the prevalence of unrecognized heart failure (HF-REF, HF-PEF, and isolated right sided heart failure) in elderly who present themselves with shortness of breath on exertion to the general practitioner.

– To examine the effect of treatment guided by a structured up-titration scheme on functionality, health-related quality of life, and uptake of heart failure medication in patients with newly, screen-detected HF.

– To assess the cost-effectiveness of such an intervention.

## Methods/Design

### Study design

**Figure 1:**
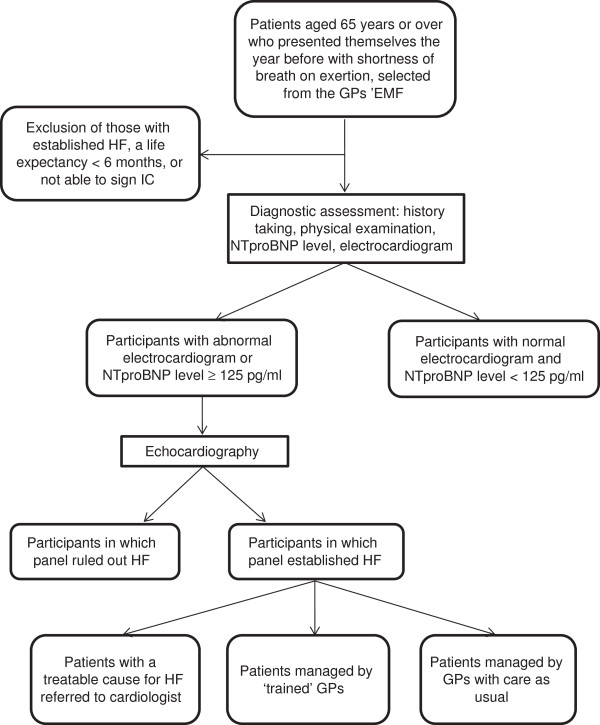
**Flowchart STRETCH – study.** GP = general practitioner, EMF = electronic medical file, HF = heart failure, IC = informed consent, NTproBNP = N-terminal pro B-type natriuretic peptide.

Our design consists of two parts. The first part is a selective screening study (case finding) of HF in elderly with shortness of breath on exertion. The second part is a single-blind, cluster randomized controlled trial comparing an intervention at the level of the GP to improve treatment uptake with care as usual in those with screen-detected HF. The intervention consists of a half-day training in the management of HF, and the practical application of an up-titration scheme for HF-REF and HF-PEF separately. See Figure 1 for the study flowchart.

### Study population and recruitment

Thirty primary care practices in the Zeist region, located in the vicinity of the Diakonessenhuis hospital Zeist, the Netherlands, participate in this study. In this sample of primary care practices, urban, suburban and rural communities are represented.

The study population consists of persons aged 65 years or over who in the previous 12 months presented themselves to one of the participating GPs with shortness of breath on exertion (including those known with a pulmonary disease), irrespective of whether they were suspected of HF or not. Shortness of breath was not necessarily the main reason for contact. Eligible subjects are identified from the electronic medical files of the participating GPs by a single physician (EvR).

Patients already known with an established diagnosis of HF confirmed by a cardiologist with help of an echocardiography are excluded, as are patients with a life expectancy shorter than 6 months, and those unable to give informed consent.

We consider our study sample representative for elderly aged 65 years or over in the community, since in the Netherlands all citizens, except those living in a nursing home or hospice, are registered with a GP, irrespective of (co)treatment by a specialist.

### Randomization and blinding

Random allocation to either management guided by a structured up-titration scheme (intervention group) or care as usual (control group) will be executed at the level of the GP. As a result, patients with HF of one and the same GP (a cluster) will all be managed according to the same arm. This approach was taken to reduce the risk of contamination between patient groups.

Since it is not deemed feasible to keep the GPs in the care as usual group unaware of the existence of an intervention arm, blinding of the participants is considered necessary to minimize bias in assessing the outcome parameters. In addition, the researcher performing the six minutes walking test is blinded to the patient’s allocation arm. The risk of observer bias for the other endpoints is minimized, because questionnaires on quality of life are filled out at home by the participants themselves, and retrieving information on HF medication and HF related doctor appointments from the electronic medical files is not subject to interpretation.

### Sample size

The difference in mean distance walked during the six-minute walking test (6MWT) between the two groups after six months follow-up will be taken as the primary outcome. The average distance walked by HF patients ranges from 242 m (standard deviation 71 m) up to 427 m (standard deviation 100 m), depending on the severity of HF [13]. Since it was shown that an increase of 50 m in walking distance indicated a clinically relevant change [14,15], we consider a mean difference of 50 meters between groups as the minimal important difference to show an effect of the intervention.

We used the two-sample t-test power analysis to examine how many HF patients are needed per group to demonstrate an effect. With an effect size of 0.6, alpha of 0.05, and beta of 0.2, group sample sizes of two times 45 will be sufficient.

We assume that in a standard general practice with 2400 patients, at least 50 patients aged 65 years or over will experience shortness of breath during a one-year period. The prevalence of previously unrecognized HF in such patients is expected to be around 14% (10.4-17.5%) [16]. With a participation rate of 50%, a mean number of 3.5 patients per GP are expected to have newly detected HF (50% HF-REF and 50% HF-PEF). Twenty-six general practitioners are needed to reach the targeted number of HF patients. We will include 4 extra practices to allow for taking clustering into account.

### Diagnostic procedures

Participants will be send two health-related quality of life (HRQoL) questionnaires. They will fill out these questionnaires before the initial baseline assessment that will be performed by a trained physician and research nurse at the outpatient clinic of the Julius Center. If unable to travel to the study center, participants will be visited at home by the physician. Informed consent will be obtained from all participants.

After signing informed consent, participants receive a diagnostic work-up scheme. Diagnostic investigations include history taking, physical examination, an electrocardiogram (ECG), and a blood test for N-terminal pro B-type natriuretic peptide (NTproBNP) levels. For the assessment of medical history, comorbidities, symptoms, and current drug use, a standardised digital questionnaire will be used. Physical examination includes height and weight, blood pressure (two readings), pulse, respiratory rate, pulmonary percussion and auscultation, heart auscultation, palpation of the apex beat, measurement of the jugular venous pressure, palpation of the liver and inspection for signs of venous insufficiency.

Blood serum concentrations of NTproBNP will be analysed with a non-competitive immunoradiometric assay (Roche, Mannheim, Germany). NTproBNP is considered elevated if the value exceeds the exclusionary cut point 15 pmol/L (~125 pg/ml), in line with the ESC guidelines on HF [17].

A standard 12-lead ECGs will be recorded in supine position. All ECGs will be analysed by a trained GP with special expertise in heart failure (FR), according to the Minnesota coding criteria [18], without knowledge of the patients’ clinical status.

The ECG is considered abnormal when one of the following is present; atrial fibrillation, sinus tachycardia (heart rate > 100 beats per minute), left and right bundle branch block (complete and incomplete), left anterior and posterior block, left ventricular hypertrophy, pathological Q-waves suspected for previous myocardial infarction, P-wave abnormalities compatible with left atrial enlargement, ST-segment/T-wave abnormalities, or second or third degree AV block.

Only participants with an abnormal ECG or an elevated blood NTproBNP level will undergo additional echocardiography in the outpatient clinic of the Diakonessenhuis Hospital Zeist. Echocardiography is performed by a single trained and experienced cardiac sonographer (HvdH) using a Philips iE33 imaging system, Andover MA, blinded to the patients’ test results of the diagnostic work-up. Guidelines of the American Society of Echocardiography [19,20] will be followed to assess structural and functional cardiac properties. The left ventricular ejection fraction (LVEF) will be assessed quantitatively, or semi-quantitatively when necessary. Multiple diastolic parameters will be measured, including pulsed-wave Doppler of the mitral and pulmonary venous inflow and tissue Doppler imaging of the mitral annulus motion. The ratio of peak early (E) diastolic filling velocity to peak of atrial (A) contraction (E/A ratio) will be calculated. The early diastolic mitral annular velocity (e’) will be determined at the septal and lateral wall, and the average of both velocities will be used to calculate the E/e’ ratio.

Participants will register the experienced burden of the aforementioned investigations on a visual analogue scale (VAS) (0 = not burdening at all, 10 = extremely burdening).

The participating GPs will receive a first preliminary conclusion about the presence or absence of HF, based on all available information from the diagnostic work up. GPs will be asked to initiate treatment in patients with HF according to their allocation (intervention or care as usual). An expert panel consisting of two cardiologists and a GP will finally establish or exclude HF.

Participants with a newly established diagnosis of HF will be asked to perform a 6MWT [21], and again after six months of follow-up. All participants will be asked to fill out the HRQoL questionnaires again six months after the diagnostic assessment.

In addition, information about medication, HF related appointments with the GP, HF related referrals to the cardiologist, and HF related hospitalization rates will be extracted from the electronic medical files at the GP’s office during 12 months of follow-up.

### Intervention

Patients with newly diagnosed HF will participate in the cluster randomize trial, except for those having a potentially treatable cause of their HF; they will immediately be referred to a cardiologist. All other HF patients will receive either care as usual or management guided by a structured up-titration scheme (intervention) by their own GP.

The GPs randomly allocated to the intervention group will receive a half-day training in the management of HF and the practical application of an up-titration scheme for HF-REF and HF-PEF separately, based on the Dutch GPs’ heart failure guideline (NHG-standaard Hartfalen 2005) [22], a Dutch equivalent of the recent ESC Guideline on heart failure [4].

In patients with newly established HF-REF, the up-titration scheme will start with a combination of a diuretic and an ACE-inhibitor, or in case of intolerance to an ACE-inhibitor an ARB. In case of acceptable blood pressures, ACE-inhibitors will be further up-titrated until half of the recommended dose. When patients are in euvolemic state and there are no contra-indications, GPs will then start with up-titration of a beta-blocker. The dosage of the beta-blocker will be increased to either the maximal recommended dose or the maximal tolerated dose. After the up-titration period of the beta-blocker, the GP will try to further increase the dosage of the ACE-inhibitor up to either the maximal recommended dose or maximal tolerated dose.

**Figure 2:**
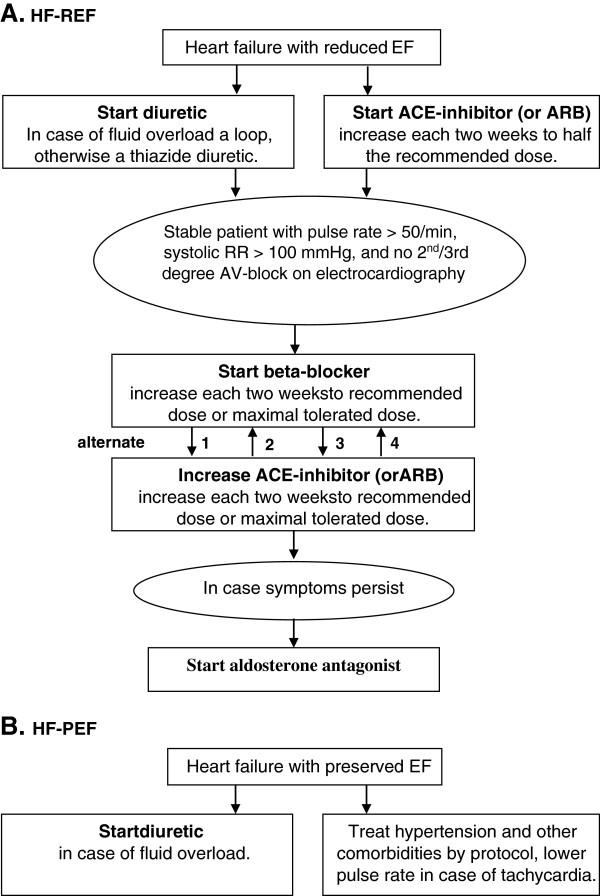
**Simplified version of the treatment scheme for heart failure.** HF-REF = heart failure with reduced ejection fraction, HF-PEF = heart failure with preserved ejection fraction, EF = ejection fraction, ACE = angiotensin converting enzyme, ARB = angiotensin receptor blocker, min = minute, RR = blood pressure, ECG = electrocardiogram, AV-block = atrioventricular block.

Patients with newly established HF-PEF will receive optimal blood pressure treatment. An ACE-inhibitor (or ARB) or low dosages of diuretics will be prescribed. In case of tachycardia such as in atrial fibrillation, the GP will aim to regulate the heart rate, preferably with a beta-blocker. A diuretic will also be prescribed if signs or symptoms of fluid or salt retention are present. See Figure 2 for a simplified version of the initiation- and up-titration scheme for HF patients, and Additional file 1 for the full content of the scheme.

The GPs randomly allocated to the control group will manage patients with a newly screen-detected diagnosis of HF as they are used to (care as usual). They are expected to follow the same Dutch heart failure guideline, however, they will lack the guided up-titration scheme and will not receive a special training.

Referral to a cardiologist is possible at any stage in this pragmatic trial. GPs in both groups will receive instructions about when it is advisable to refer patients for specialist consultation in line with the guidelines on HF, e.g. if patients remain symptomatic after the up-titration phase, in case of complications or adverse effects, and when a novel treatable cause of HF is suspected.

### Outcome measures

#### Heart failure

An expert panel, consisting of two cardiologists (AL and ML) and one GP with special expertise in heart failure (FR) will determine the presence or absence of HF based on all diagnostic test results. Consensus diagnosis by an outcome panel is the preferred method in case a sufficiently reliable reference standard is lacking, as is the case for HF [7].

The panel moves along three phases in their decision making process; part 1 history taking and physical examination, part 2 adding NTproBNP and ECG, and part 3 adding echocardiography. After part 1 and 2, the panel expresses their suspicion for the presence of HF in a percentage (0-100%). With this approach, we can evaluate which investigations add most in reaching the final decision (set in part 3).

To classify participants as (not) having HF, the panel will follow the latest criteria of the European Society for Cardiology [17]. HF is considered present when participants have suggestive symptoms (typically breathlessness at rest or on exercise, fatigue, tiredness, ankle swelling) and signs (typically tachycardia, tachypnea, pulmonary rales, raised jugular venous pressure, peripheral oedema, laterally displaced apical beat) in combination with objective echocardiographic evidence of cardiac dysfunction at rest. Signs of volume overload could be masked when diuretics are used for hypertension, and therefore in participants on diuretics signs of fluid overload are not mandatory.

Heart failure is further classified in HF-REF, HF-PEF, or isolated right sided HF. Patients in whom signs and symptoms of HF are present in combination with a LVEF ≤ 45%, are classified as having HF-REF. Patients in whom signs and symptoms of HF are present in combination with a LVEF > 45% and echocardiographic evidence of diastolic dysfunction, are classified as having HF-PEF.

The E/e’ ratio is regarded as a key parameter for assessing diastolic dysfunction; an E/e’ greater than or equal to 15 is considered abnormal, an E/e’ value between 9 and 14 indeterminate, and an E/e’ less than 9 as normal. In case the E/e’ is indeterminate, at least two additional abnormal parameters are needed to establish diastolic dysfunction [20,23]; an E/A ratio below 0.75 or above 1.5, an E-deceleration time >280 ms, a time difference between the duration of the atrial reversal velocity and the late diastolic mitral inflow duration (Ar - A) of more than 30 ms, a left atrial volume index > 34 ml/m^2^, a left ventricular mass indexed by body surface area > 95 g/m^2^ in women, and > 115 g/m^2^ in men (according to the formula of Devereux [24].

A complete assessment of diastolic dysfunction is not possible in participants with atrial fibrillation. In such participants an elevated indexed left atrial volume (≥34 ml/m^2^) in combination with AF will be considered sufficient to establish diastolic dysfunction.

To diagnose isolated right-sided HF, patients need to have signs and symptoms of HF in combination with a LVEF > 45%, and an increased pulmonary artery pressure (calculated systolic pulmonary artery pressure >40 mmHg), in the absence of (overt) diastolic dysfunction.

By re-assessment of a random sample of 10% by the same outcome panel, blinded to the original decision, the reproducibility of the panel diagnoses will be evaluated.

#### Functional capacity

For the elderly HF patient exercise intolerance can be very limiting and of major clinical importance. We chose the 6MWT for the objective evaluation of submaximal functional capacity in patients with newly established HF, because the exercise level is consistent with daily physical activities [21]. The 6MWT is considered a valid, easy, well-tolerated, and inexpensive test [25] that measures the distance that a patient can walk on a flat, hard surface during 6 minutes. The test will be executed according to the guidelines of the American Thoracic Society [21].

#### Health-related quality of life

The SF-36 [26,27] and EQ-5D [28,29] were chosen as instruments to measure general health status of all participants. In addition, the scores of the EQ-5D will be converted to a time trade-off utility score to calculate quality-adjusted life years for the cost-effectiveness analysis.

The MLHF questionnaire was added as a condition-specific measurement for HF patients to evaluate the patients’ perception of the effects of HF on the physical, socioeconomic and psychological aspects of their life. All questionnaires are well validated and widely used.

#### Costs

Relevant cost parameters within and outside the healthcare system will be collected prospectively during a follow-up period of 12 months. Direct costs within the health care system will be monitored through the electronic medical patients files of the GPs, including the costs of further diagnostic testing, time consumption of health care workers (i.e. GP contacts, referrals to medical specialists, hospital admissions), and treatment expenses. Costs outside the healthcare system include the estimation of time-investment and travelling expenses patients make. As the population under study is older than 65 years of age, costs of productivity loss will not be studied. Regarding the standardization of costs, we will use a uniform costing methodology [30].

### Statistical analyses

Prevalence rates of newly established HF-REF, HF-PEF and isolated right sided HF in our population with shortness of breath on exertion will be calculated as proportions with 95% confidence intervals.

Differences in functionality and quality of life after 6 months of follow-up, and uptake of HF medication between both groups after 6 and 12 months of follow-up will be compared taking into account potential difference of relevant parameters at baseline (ANCOVA), although such differences are expected to be minimal because of the randomization procedure. This will be done separately for patients with HF-REF and HF-PEF. A multilevel approach is used in the analyses to correct for the fact that we randomized at the level of the GP and not at the patient level.

The cost-effectiveness of training GPs in the management of HF and the practical application of an up-titration scheme will be evaluated and compared with care as usual. Cost-effectiveness will be expressed in terms of costs per quality-adjusted life year gained (cost-utility analysis). Estimates have to be made in the calculation of the costs and the definition of effects, and these are subjected to different margins of uncertainty. To assess to what extent the results of the analysis depend on prior assumptions and choices made, a sensitivity analysis will be performed conform current Dutch standards for pharmacoeconomic research [31].

### Results of inclusion

Thirty GPs are included in the study, 14 were randomized to the care as usual arm and 16 to the intervention arm. We invited 1,527 elderly patients with shortness of breath on exertion to participate in the study. Of the invited patients, 77% responded, and eventually 38% was willing to participate. Reasons named for non-participation were; expected burden too high (40%), already known to a cardiologist (24%), other demanding health problems (15%), not experiencing shortness of breath anymore (12%), and severe immobility (5%).

Finally, 585 patients signed informed consent and were included in our study. The mean age of the participants was 74.1 (SD ±6.3) years. Stratified by age, 57% was aged between 65 and 74 years, 37% between 75 and 84 years, and 6% was aged 85 years or over. The percentage females was 54.5%. Important comorbidities were pulmonary disease (55.2%), hypertension (53%), hypercholesterolemia (32%), and ischemic heart disease (20%). The 14 GPs in the care as usual arm yielded 351 participants, and the 16 GPs of the up-titration arm 234 participants. Baseline characteristics of included patients divided between both groups are described in Table 1.

**Table 1:** Baseline characteristics of the 585 participants, divided in the intervention and care as usual group

**Characteristics**	**All**	**Care as usual**	Intervention*	
	**n = 585**	**n = 351 (60%)**	**n = 234 (40%)**	**p-value**
General				
Mean age in years ± sd	74.1 ± 6.3	73.8 ± 6.1	74.4 ± 6.5	0.25
Female sex	319 (54.5)	181 (51.6)	138 (59.0)	0.08
MRC dyspnea score ≥ 3	157 (26.8)	97 (27.6)	60 (25.6)	0.59
Cardiovascular comorbidities				
Ischemic heart disease	116 (19.8)	70 (19.9)	46 (19.7)	0.93
Prior myocardial infarction	43 (7.4)	27 (7.7)	16 (6.8)	0.70
Prior PCI/CABG	47 (8.0)	29 (8.3)	18 (7.7)	0.80
Vascular comorbidity	351 (60.0)	217 (61.8)	134 (57.3)	0.27
Hypertension	310 (53.0)	196 (55.8)	114 (48.7)	0.09
Hypercholesterolemia	187 (32)	115 (32.8)	72 (30.8)	0.61
Diabetes Mellitus	79 (13.5)	47 (13.4)	32 (13.7)	0.92
Prior stroke or TIA	51 (8.7)	27 (7.7)	24 (10.3)	0.28
Peripheral arterial disease	35 (6.0)	22 (6.3)	13 (5.6)	0.72
Atrial fibrillation	42 (7.2)	33 (9.4)	9 (3.8)	0.01
Non-cardiovascular comorbidities				
Cognitive disorders	110 (18.8)	50 (14.2)	60 (25.6)	0.001
Asthma/COPD	323 (55.2)	200 (57.0)	123 (52.6)	0.29
Depression	72 (12.3)	39 (11.1)	33 (14.1)	0.28
Medication				
Loop diuretics	55 (9.4)	36 (10.3)	19 (8.1)	0.39
Thiazide diuretics	147 (25.1)	89 (25.4)	58 (24.8)	0.88
ACE-inhibitors	120 (20.5)	70 (19.9)	50 (21.4)	0.68
ARBs	128 (21.9)	83 (23.6)	45 (19.2)	0.21
Beta-blockers	128 (21.9)	79 (22.5)	49 (20.9)	0.65
Digitalis	5 (0.9)	4 (1.1)	1 (0.4)	0.36
Aldosterone antagonists	7 (1.2)	7 (2.0)	0 (0.0)	0.03
Oral anticoagulants	51 (8.7)	37 (10.5)	14 (6.0)	0.06
Antiplatelets	147 (25.1)	84 (23.9)	63 (26.9)	0.41
Statins	179 (30.6)	110 (31.3)	69 (29.5)	0.63
Nitrates	41 (7.0)	26 (7.4)	15 (6.4)	0.64
Calcium channel blockers	76 (13.0)	54 (15.4)	22 (9.4)	0.04

Values are numbers (percentages).

*Intervention = a half-day training in the management of HF and the practical application of an up-titration scheme for HF-REF and HF-PEF.

Sd = standard deviation, MRC = medical research council, PCI = percutaneous coronary intervention, CABG = coronary artery bypass grafting, TIA = transient ischemic attack, COPD = chronic obstructive pulmonary disease, ACE = angiotensin converting enzyme, ARB = angiotensin receptor blocker, HF = heart failure, HF-REF = heart failure with reduced ejection fraction, HF-PEF = heart failure with preserved ejection fraction.

## Discussion

The diagnostic part of our study will provide information on the prevalence rate of previously unrecognized HF in older community-dwelling persons with shortness of breath on exertion. The cluster randomized comparison will reveal whether a single half-day training of GPs in the management of HF and the practical appliance of an up-titration scheme results in subsequent improvements in functionality, HRQoL, and uptake of HF medication compared to care as usual. In addition, cost-effectiveness will be examined. We expect that our diagnostic-therapeutic strategy is easy applicable and implementable, and above all, is able to detect a large number of patients with previously unrecognized HF in primary care and optimize their treatment, resulting in increased functional capacity and HRQoL.

### Regulation statement

This study is conducted according to the principles of the current version of the declaration of Helsinki and in accordance with the Dutch law on Medical Research Involving Human Subjects Act (WMO).

### Ethics committee approval

The study was approved by the medical ethical committee (METC) of the University Medical Center Utrecht, the Netherlands.

## Abbreviations

HF: Heart failure; HF-REF: Heart failure with reduced ejection fraction; ACE: Angiotensin converting enzyme; ARB: Angiotensin receptor blocker; HFPEF: Heart failure with preserved ejection fraction; GP: General practitioner; 6MWT: Six-minute walking test; HRQoL: Health-related quality of life; ECG: Electrocardiogram; NTproBNP: Amino-terminal pro-B-type natriuretic peptide; LVEF: Left ventricular ejection fraction; VAS: Visual analog scale; SF-36: Medical outcomes study 36-item short form health survey; EQ-5D: EuroQol; ANCOVA: Analysis of covariance.

## Competing interests

All authors declare no competing interests.

## Authors’ contributions

AW and FH designed the study. FH coordinated the study. EvR managed the study and data collection. HvdH performed all echocardiographies. AL, ML and FH were members of the expert panel. EvR conducted the data analyses and wrote the first draft of the manuscript. All authors read and approved the final draft of the manuscript.

## Pre-publication history

The pre-publication history for this paper can be accessed here:

http://www.biomedcentral.com/1471-2261/14/1/prepub

## Supplementary Material

Additional file 1 **Initiation- and up-titration scheme for patient with newly, screen-detected HF.** Scheme handed to participating GPs to facilitate easy initiation and up-titration of heart failure medication in patients with newly, screen-detected HF. Also includes contra-indications of medications, instructions for common barriers experienced during up-titration, and a reminder for periodic check-ups. Click here for file
