# Supplementary material for: Strategy to recognize and initiate treatment of chronic heart failure in primary care (STRETCH): a cluster randomized trial
Source: BMC Cardiovasc Disord. 2014 Jan 8;14:1. doi: 10.1186/1471-2261-14-1 (PMC3898002; doi:10.1186/1471-2261-14-1)
Supplement: Additional file 1 — Initiation- and up-titration scheme for patient with newly, screen-detected HF. Scheme handed to participating GPs to facilitate easy initiation and up-titration of heart failure medication in patients with newly, screen-detected HF. Also includes contra-indications of medications, instructions for common barriers experienced during up-titration, and a reminder for periodic check-ups. [file 1471-2261-14-1-S1.doc]

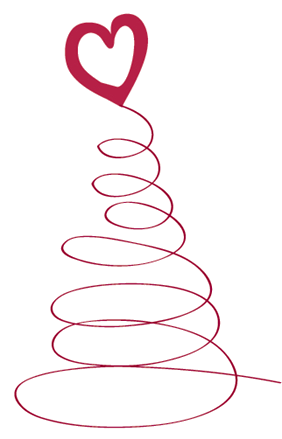


**CONTRA-INDICATIONS**

**FLOWCHART**

Heart failure with reduced EF

**ACE-inhibitor**

- Angioedema in history

- Bilateral renal artery stenosis

- Sodium > 5.0 mmol/l

- Creatinine > 220 μmol/l

- Severe aortic valve stenosis

**ARB**

- Same as ACE-inhibitor except for angioedema

- Combination with ACE-inhibitor and aldosterone antagonist

**Beta-blocker**

- Asthma (COPD is not a contra-indication!)

- 2^nd^ of 3^rd^ degree AV-block, sinus bradycardia and sick sinus syndrome

**Aldosterone antagonist**

- Sodium > 5.0 mmol/l

- Creatinine > 220 μmol/l

- Concurrent use of potassium-sparing diuretic or potassium suppletion

- Combination with ACE-inhibitor and ARB

**NSAIDs** should preferably be avoided

A

**Start diuretic** in case of fluid overload a loop, otherwise a thiazide dieretic.

**Start beta-blocker** increase each two weeks to recommended dose or maximal tolerated dose.

**Start aldosterone antagonist**

alternate

1

2

3

4

B

|  | **Diuretic** | | **ACE-inhibitor** | | **Beta-blocker** | |
| --- | --- | --- | --- | --- | --- | --- |
|  | ↑ | ↓ | stop | 1/2 | ↓ | not ↑ |
| **Sodium > 5.5 mmol/l** |  |  |  | ● |  |  |
| **Sodium > 6.0 mmol/l** |  |  | ● |  |  |  |
| **MDRD < 30 ml/min** |  |  |  | ● |  |  |
| **MDRD < 10 ml/min** |  |  | ● |  |  |  |
| **Dizziness** |  |  |  |  |  | ● |
| **↑** **edema** | ● |  |  |  |  | ● |
| **Syst. RR < 100 mm Hg** |  | ● |  |  |  | ● |
| **Clinical ‘detoriation’** | ● |  |  |  | ● |  |
| **Pulse rate < 50/min** |  |  |  |  | ECG |  |

**WHAT TO DO IF..**

**STRETCH**

**CONTACT**

Dr. F.H. Rutten

Tel:

F.H.Rutten@umcutrecht.nl

Drs. S. Limburg

Tel:

slimburg@diakhuis.nl

Drs. E.E.S van Riet

Tel:

E.E.S.vanRiet@umcutrecht.nl

**Start ACE-inhibitor (or ARB)** increase each two weeks to half the recommended dose.

**Increase ACE-inhibitor (or ARB)** increase each two weeks to recommended dose or maximal tolerated dose.

**Start diuretic** in case of fluid overload.

Treat hypertension and other comorbidities by protocol, lower pulse rate in case of tachycardia.

Heart failure with preserved EF

**PERIODIC CHECK-UPS and UP-TITRATION PROTOCOL**

| **1. Diuretics** | **Starting dose** | **Usual dose** |
| --- | --- | --- |
| ● Furosemide | 20 mg qd | 40-120 mg qd |
| ● Bumetanide | 0.5 mg qd | 1-5 mg qd |
| ● Hydrochloorthiaz. | 12.5 mg qd | 12.5-100 mg qd |
| ● Chloortalidon | 25 mg qd | 12.5-50 mg qd |

|  | **2 weeks** | **4 weeks** | **6 weeks** | **8 weeks** | **10 weeks** | **12 weeks** | **After reaching maximal tolerated dose or recommended dose: on month 1, 2, 3 and 6 and thereafter each 6 months** |
| --- | --- | --- | --- | --- | --- | --- | --- |
| Sodium, Potassium | ● |  | ● |  | ● |  | ● |
| Creatinine, MDRD | ● |  |  |  |  |  | ● |
|  |  |  |  |  |  |  |  |
| Adverse drug reaction | ● | ● | ● | ● | ● | ● | ● |
| Weight | ● | ● | ● | ● | ● | ● | ● |
| Pulse rate, RR | ● | ● | ● | ● | ● | ● | ● |
| Oedema, Rales | ● | ● | ● | ● | ● | ● | ● |

|  | **Starting dose** | **2 weeks** | **4 weeks** | **6 weeks** | **8 weeks** | **10 weeks** | **12 weeks** | **Recommended dose** |
| --- | --- | --- | --- | --- | --- | --- | --- | --- |
| **2a. ACE-inhibitors** |  |  |  |  |  |  |  |  |
| ● Enalapril | 2.5 mg qd | 2.5 mg qd | 5 mg qd | 7.5 mg qd | 10 mg qd |  |  | 10-20 mg bid |
| ● Lisinopril | 2.5 mg qd | 2.5 mg qd | 5 mg qd | 10 mg qd | 20 mg qd | 30 mg qd |  | 20-35 mg qd |
| ● Captopril | 6.25 mg tid | 12.5 mg tid | 25 mg tid | 37.5 mg tid | 50 mg tid |  |  | 50-100 mg tid |
| ● Ramipril | 2.5 mg qd | 2.5 mg bid | 5 mg bid |  |  |  |  | 5 mg bid |
| ● Fosinopril | 5 mg qd | 10 mg qd | 20 mg qd | 40 mg qd |  |  |  | 20-40 mg qd |
|  |  |  |  |  |  |  |  |  |
| **2b. ARBs** |  |  |  |  |  |  |  |  |
| ● Losartan | 50 mg qd | 100 mg qd |  |  |  |  |  | 100 mg qd |
| ● Candesartan | 4 mg qd | 8 mg qd | 16 mg qd | 32 mg qd |  |  |  | 32 mg qd |
| ● Valsartan | 40 mg bd | 80 mg bd | 160 mg bd |  |  |  |  | 160 mg bid |
|  |  |  |  |  |  |  |  |  |
| **3. Beta-blockers** |  |  |  |  |  |  |  |  |
| ● Bisoprolol | 1.25 mg qd | 2.5 mg qd | 3.75 mg qd | 5.0 mg qd | 5.0 mg qd | 7.5 mg qd | 10 mg qd | 10 mg qd |
| ● Metoprolol succ. | 12.5 mg qd | 25 mg qd | 50 mg qd | 100 mg qd | 150 mg qd | 200 mg qd |  | 200 mg qd |
| ● Nebivolol | 1.25 mg qd | 2.5 mg qd | 5 mg qd | 10 mg qd |  |  |  | 10 mg qd |
| ● Carvedilol | 3.125 mg bid | 6.25 mg bid | 12.5 mg bid | 25 mg bid | (37.5 mg bid) | (50 mg bid) |  | <85 kg: 25 mg bid  >85 kg: 50 mg bid |
|  |  |  |  |  |  |  |  |  |
| **4. Aldosterone antagonists** | |  |  |  |  |  |  |  |
| ● Spironolacton | 25 mg qd |  |  |  |  |  |  | 25 mg qd |
| ● Eplerenone | 25 mg qd | 25 mg qd | 50 mg qd |  |  |  |  | 50 mg qd |

Heart failure with preserved LVEF

**Start Diuretic** In case of edema a loop diuretic, otherwise a thiazide diuretic.

Treat hypertension and myocardial infarction by protocol. Pulse rate↓ but > 50/min.

Heart failure with preserved LVEF

**Start Diuretic** In case of edema a loop diuretic, otherwise a thiazide diuretic.

Treat hypertension and myocardial infarction by protocol. Pulse rate↓ but > 50/min.

Heart failure with preserved LVEF

Heart failure with preserved LVEF
